# Effectiveness and Acceptability of Visual Inspection With Acetic Acid as a Screening Test for Cervical Intraepithelial Neoplasia

**DOI:** 10.7759/cureus.99960

**Published:** 2025-12-23

**Authors:** Mathanky Theeparuban, Muhunthan Kopalasuntharam

**Affiliations:** 1 Department of Obstetrics and Gynaecology, Teaching Hospital Jaffna, Jaffna, LKA; 2 Obstetrics and Gynecology, Faculty of Medicine, University of Jaffna, Jaffna, LKA

**Keywords:** accuracy of via, cervical screening, cin, colposcopy, via, visual inspection of cervix

## Abstract

Introduction: Cervical carcinoma is a significant burden in developing countries, and it is preventable by early detection of its precursor lesions through screening.

Aim and objectives: This study assessed the effectiveness and acceptability of visual inspection of the cervix with acetic acid as a screening test in a low-resource setting, comparing it with colposcopy-guided biopsy as a gold standard test. It also assessed the prevalence of preneoplastic conditions of the cervix and its association with known risk factors for cervical carcinoma.

Materials and methodology: A comparative study was conducted among 978 women in the age group of 35-54 years from Velanai MOH area in Jaffna, Sri Lanka, during the period of April 2017 to March 2018. The interviewer administered a questionnaire, Visual Inspection of Cervix with Acetic acid (VIA), and colposcopy reporting forms were used for data collection. All participants were screened with VIA and then subjected to colposcopy immediately. Targeted biopsies were performed in 107 cases. Effectiveness of VIA was assessed by means of sensitivity, specificity, positive predictive value, and negative predictive value. Chi-squared test and Fisher’s exact test were used to assess the association of risk factors with cervical intraepithelial neoplasia (CIN) and the acceptability of the screening test.

Results: The prevalence of preneoplastic conditions, including CIN 1, during the study period of April 2017 to March 2018, was 3.75% in the study population. Early sexual activity, usage of combined oral contraceptive pills, and multiparity were significantly associated with CIN 2 or more lesions (P<0.05). 97.86% of women stated that they would recommend cervical screening with VIA to others. Only six women (0.64%) had heavy bleeding from the biopsy, and 71 women (7.6%) reported severe pain. In detecting CIN 2 or more, the sensitivity of VIA was 70.6%, the specificity was 74.7%, the positive predictive value was 4.9%, and the negative predictive value was 99.3%. When considering the performance of VIA and colposcopy, colposcopy performed well at detecting CIN 2 or more lesions.

Discussion: Even though the sensitivity, specificity, and negative predictive value of VIA were high, it has a low positive predictive value. VIA can be used as an alternative screening test in young women in low-resource settings like Sri Lanka. But it should be kept in mind that the higher false positive rate may result in unnecessary interventions and complications, and it is not suitable for older women with a non-visible transformation zone.

## Introduction

Cervical carcinoma is the eighth most common cancer among women throughout the world, and about 60% of these cases occur in Asian countries [1]. It is the second most common cancer in females in Sri Lanka, with 1407 women diagnosed in 2023 with a crude incidence rate of 12.5/100,000 females per year [2]. Squamous cell carcinoma is the most common type (80-90%) [3], which arises from precursor lesions called Cervical Intraepithelial Neoplasia (CIN). CIN is caused by infection of the cervix with Human Papillomavirus (HPV), which is transmitted by sexual contact. Infection with high-risk HPV subtypes leads to the development of CIN, which can progress into invasive cervical carcinoma if not treated [4-6]. HPV acts on immature metaplastic squamous cells in the transformation zone of the uterine cervix and induces dysplastic changes that range from CIN 1, CIN 2, CIN 3 and invasive cancer.

There are several risk factors for infection with HPV, such as early sexual activity (<16 years), multiple sexual partners, and multiple sexual contacts of her partner [7]. Infection with oncogenic HPV types is not sufficient for the development of cervical cancer. Presence of other cofactors such as active or passive smoking [8], immunosuppression, and co-infection with Human Immunodeficiency Virus (HIV) [9], and presence of other sexually transmitted infections increase the risk of developing invasive cervical cancer. Long-term use of hormonal contraceptives and multiparity increase the risk of cervical cancer by exposing the columnar epithelium to the vaginal milieu and HPV infection. 

Cervical intra-epithelial neoplasia is mostly asymptomatic but can be detected by cervical screening tests such as Papanicolaou smear (PAP), liquid-based cytology (LBC), Visual Inspection of cervix with Acetic acid (VIA), Visual Inspection with Lugol’s Iodine (VILI), high-risk HPV DNA/ E6 & E7 mRNA/ onco-proteins testing, and other novel methods [10]. In developed countries, screening with cytology and HPV DNA testing and vaccination against high-risk HPV have remarkably reduced the incidence of cervical cancer. But these tests require a well-established network, equipment, and trained human resources, and are expensive for people living in low- and middle-income countries. In contrast, VIA is less expensive and proven as cost-effective in detecting pre-neoplastic stages and early cancer in some parts of India, Thailand, and African countries [11].

As a developing country, Sri Lanka still struggles to implement the screening services. The well-women programme of the Family Health Bureau is targeting the 35 and 45-year age cohort only, and it achieved about 52% coverage for Pap smear in the 35-year age cohort in 2016, and it achieved only 31% coverage in Jaffna district [12].

VIA involves treating the cervix with 3-5% acetic acid and inspection with the naked eye for discrete whitish areas around the squamo-columnar junction after one minute. Acetic acid coagulates the cellular proteins, which are abundant in neoplastic cells [13]. The results are immediately available and depend on the severity of colour changes, patients can be treated on the same day either with cryotherapy, LEEP (Loop Electrosurgical Excision Procedures) or cold- knife conisation [14].

VIA has a comparable sensitivity (45-79% versus 47-62%) and specificity (85% versus 94%) in relation to Pap smear [11,15]. Thus, VIA can be considered as an alternative to Pap smear in low-resource countries to detect preneoplastic stages. This study assessed the acceptability and effectiveness of VIA as a screening tool for cervical preneoplastic conditions among women from a rural population in Sri Lanka with limited access to healthcare facilities due to geographic location.

## Materials and methods

This is a comparative study conducted at Divisional hospitals in Velanai MOH area by the Department of Obstetrics and Gynaecology, University of Jaffna, during the period of April 2017 to March 2018. A total of 978 sexually active women in the age group of 35-54 years were invited for this study. Women who are not consenting/not cooperative, women who had a total hysterectomy/already diagnosed to have cervical carcinoma, unsatisfactory colposcopy due to Type 3 transformation zone, and pregnant women were excluded from this study.

All women were informed about the procedure, and written consent was obtained. An interviewer-administered questionnaire was used for data collection. VIA was done by trained Intern medical officers by applying 3-5% acetic acid to the cervix, and the findings were recorded at one minute. The intensity, borders, and demarcations, location, size, and number of the aceto-white areas were documented in the IARC standard reporting form to determine the result of the VIA as positive or negative [13]. Colposcopy with 5% acetic acid & Lugol’s iodine was performed immediately by the researcher in all women irrespective of the VIA findings, and the findings were recorded separately in the IARC standard reporting form [14]. A swede score of <5 in a satisfactory colposcopy examination was considered negative, and no biopsies were done in these women. If the colposcopy result was positive, punch biopsies were taken from suspicious areas for histopathological evaluation. Those with histopathological abnormalities were treated/followed at the Teaching Hospital Jaffna.

Data analysis was done with the help of SPSS software, version 21 (IBM Corp., Armonk, NY). The accuracy of VIA was assessed using sensitivity, specificity, positive predictive value (PPV), and negative predictive value (NPV), considering colposcopy as a gold standard test. Collected data were used to determine the prevalence of CIN, the acceptability of VIA as a screening test, and the association of risk factors with these pre-neoplastic conditions.

## Results

According to Buderer's formula, the required sample size for testing diagnostic accuracy was 925. The prevalence of preneoplastic conditions was assumed to be 1% since no data were available on this topic in Sri Lanka. According to data from a study done in Egypt, the prevalence of CIN 2/3 was around 1% [16]. Among the studied sample of 978 women, 44 (4.5%) were excluded, mostly due to unsatisfactory colposcopy, resulting in a sample size of 934. According to Table 1, the mean age of participants was 45.16 years (SD=6.66), and 271 (29%) women had their last menstruation >12 months ago due to various reasons such as contraception with progesterone and menopause.

**Table 1:** Frequency distribution of age among participants

Age (Years)	Frequency	Percentage (%)
35-39	267	28.6
40-44	251	26.9
45-49	219	23.4
50-54	197	21.1
Total	934	100.0

**Figure 1:**
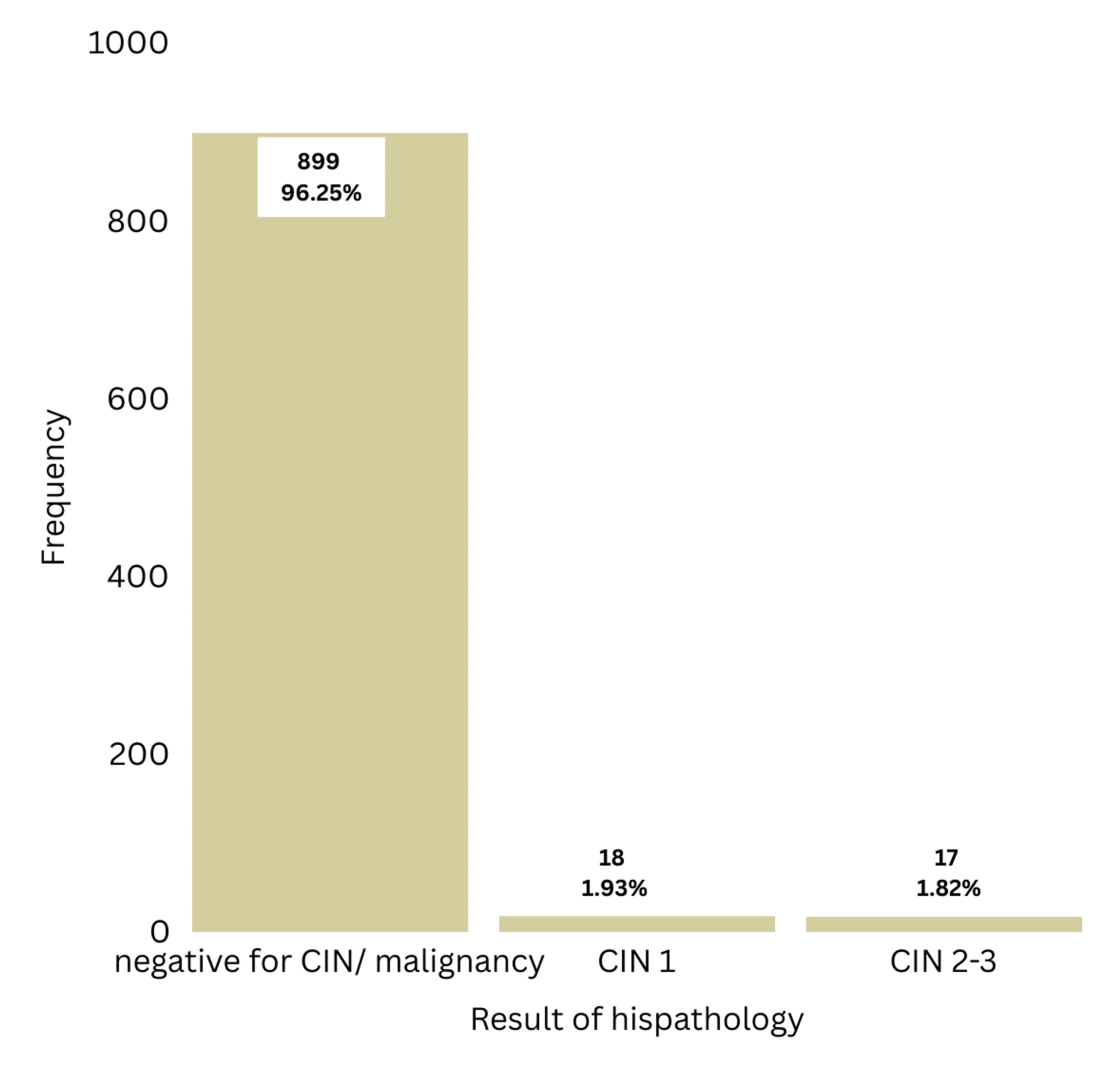
Frequency distribution of histopathology results

As per Figure 1, colposcopy-guided biopsy confirmed 18 cases of CIN 1 (1.93%) and 17 cases of CIN 2 or 3 (1.82%). Prevalence of preneoplastic conditions in the study sample was 1.93% for CIN 1 and 1.82 % for CIN 2 or CIN 3. No cases of invasive cancer were detected in this study.

**Figure 2:**
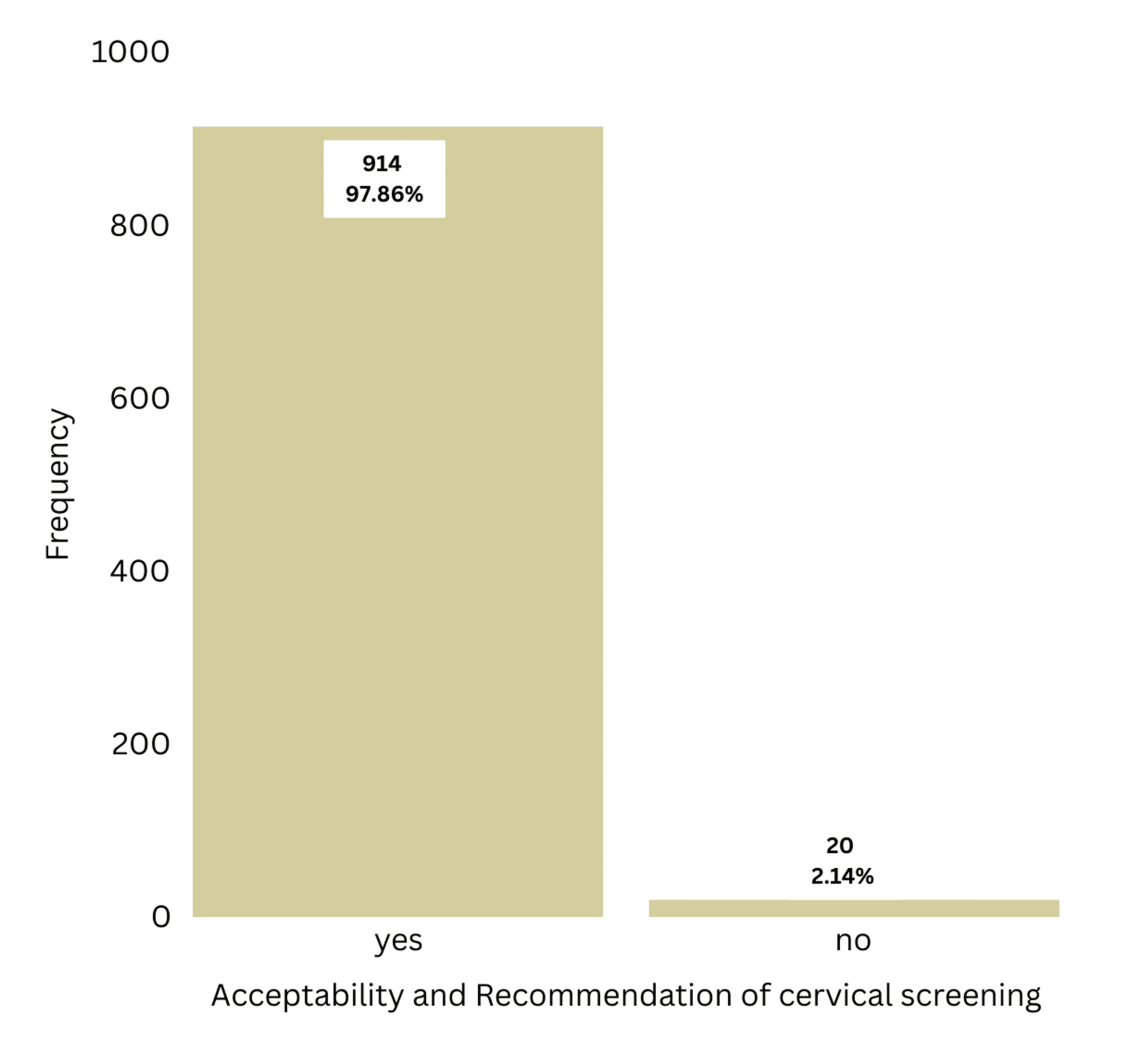
Acceptability of visual inspection with acetic acid as a screening test

**Figure 3:**
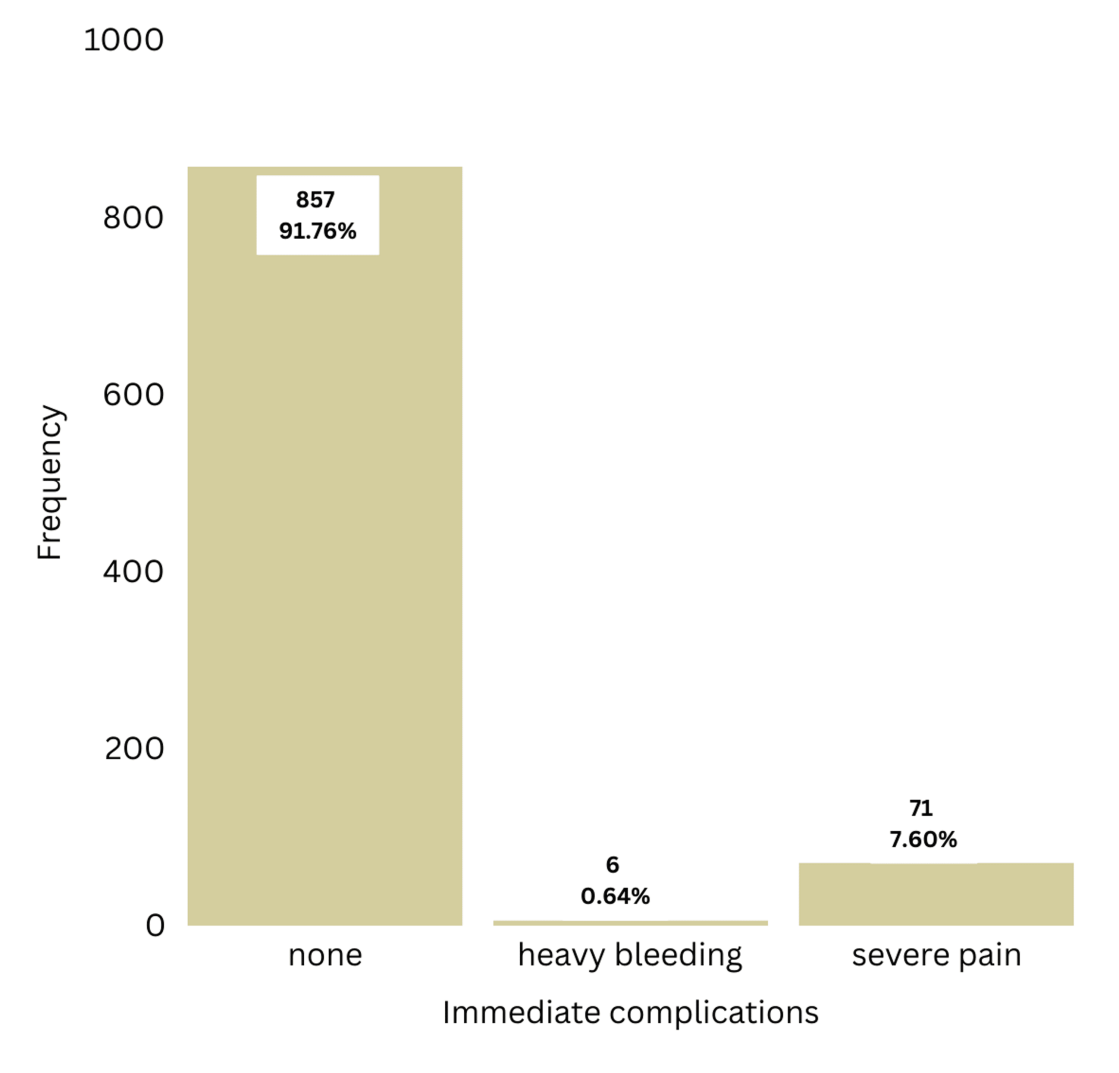
Frequency distribution of immediate complications

Per Figure 2, 914 women (97.86%) stated that they would recommend cervical screening with VIA to their relatives and friends. Only six women (0.64%) had heavy bleeding from the biopsy site, and 71 women (7.6%) stated that they had severe pain during the procedure (Figure 3).

When considering the prevalence of risk factors for cervical cancer in the study population; 113 women (12.1%) stated that they had been sexually active before the age of 18 years, 354 women (37.9%) had five or more pregnancies including miscarriages, only five women (0.53%) stated they had more than one sexual partners, 347 women (37.15%) complained of at least one symptom of STD, 186 women (19.9%) used combined oral contraceptive pills (COCP) at any time in their life and 269 women (28.8%) were exposed to passive smoking but none were active smokers. Seventy-eight women (8.3%) were on immunosuppressive treatment mainly due to poorly controlled asthma (Table 2).

**Table 2:** Prevalence and association of risk factors for cervical carcinoma with preneoplastic conditions CIN: Cervical Intraepithelial Neoplasia, STD: Sexually Transmitted Disease, COCP: Combined Oral Contraceptive Pills.

Risk factors		Result of histopathology		Total	Chi-squared test / Fisher’s exact test
		Negative for CIN	CIN 1 or more		
Age at first intercourse	< 18	103 (91.2%)	10 (8.8%)	113(100%)	χ2=9.278, df=1. p=0.002 (p<0.05)
	≥ 18	796 (97.0%)	25 (3.0%)	821 (100%)	
Number of pregnancies including miscarriages	< 5	568 (97.9%)	12 (2.1%)	580 (100%)	χ2=11.95, df=1. p=0.001 (p<0.05)
	≥ 5	331 (93.5%)	23 (6.5%)	354 (100%)	
Number of partners	1	895 (96.3%)	34 (3.7%)	929 (100%)	Fisher’s exact test. p=0.174 (p>0.05)
	≥ 2	4 (80%)	1 (20%)	5 (100%)	
Symptoms of STD	No	569 (96.9%)	18 (3.1%)	587 (100%)	χ2=2.031, df=1. p=0.154 (p>0.05)
	Yes	330 (95.1%)	17 (4.9%)	347 (100%)	
Ever usage of COCP	No	726 (97.1%)	22 (2.9%)	748 (100%)	χ2=6.768, df=1. p=0.00 (p<0.05)
	Yes	173 (93.0%)	13 (7.0%)	186 (100%)	
Smoking	No	643 (96.7%)	22 (3.3%)	655 (100%)	χ2=1.234, df=1. p=0.267 (p>0.05)
	Passive	256 (95.2%)	13 (4.8%)	269 (100%)	
Immunosuppressive treatment	No	827 (96.6%)	29 (3.4%)	886 (100%)	χ2=3.672, df=1. p=0.055 (p>0.05)
	Yes	72 (92.3%)	6 (7.7%)	78 (100%)	

When evaluating the association of risk factors with preneoplastic conditions (Table 2), early sexual activity before 18 years, ever usage of oral contraceptive pills, and five or more pregnancies, including miscarriages, were significantly associated with CIN 1 or more lesions using the chi-squared test (p<0.05). There was no significant association seen with the number of sexual partners, presence of any symptoms of Sexually Transmitted Diseases (STDs), passive smoking, or immunosuppression (p>0.05).

Among those screened, 244 women were classified as VIA positive, giving a screen-positive rate of 26.1%. When taking the cut-off of CIN 2 or more, the sensitivity of VIA was 70.6%, the specificity was 74.7%, the false positive rate was 25.3%, and the false negative rate was 29.4% (Table 3). As shown in Table 4, the positive predictive value (PPV) was 4.9%, and the negative predictive value (NPV) was 99.3.

**Table 3:** Sensitivity and specificity of VIA in detecting CIN 2 or more lesions VIA: Visual Inspection with Acetic acid, CIN: Cervical Intraepithelial Neoplasia.

			Result of Histopathology		Total
			Normal/ CIN 1	CIN 2/3	
Findings of VIA	Negative	Count	685	5	690
		% within result of histopathology	74.7%	29.4%	73.9%
	Positive	Count	232	12	244
		% within result of histopathology	25.3%	70.6%	26.1%
Total		Count	917	17	934
		% within result of histopathology	100%	100%	100%

**Table 4:** Positive and negative predictive values of VIA in detecting CIN 2 or more VIA: Visual Inspection with Acetic acid, CIN: Cervical Intraepithelial Neoplasia.

			Result of Histopathology		Total
			Normal/ CIN 1	CIN 2/3	
Findings of VIA	Negative	Count	685	5	690
		% within findings of VIA	99.3%	0.7%	100%
	Positive	Count	232	12	244
		% within findings of VIA	95.1%	4.9%	100%
Total		Count	917	17	934
		% within findings of VIA	98.2%	1.8%	100%

## Discussion

This study assessed the performance of VIA, comparing it to colposcopy with/without guided biopsy as a gold standard method in detecting CIN. As the screening test and reference test were performed on the same day, there were no cases of loss of follow-up. The age of participants ranged from 35 to 54 years. The mean age of the study sample was 45.16 years (SD=6.66). The majority (N=267) of women were in the 35-39 years age group, and the lowest frequency was observed in the 50-54 years age group (21.1%). This could be due to the higher incidence of unsatisfactory colposcopy in older women who were excluded from the study.

Prevalence of preneoplastic conditions in the study sample was 1.93% for CIN 1 and 1.82 % for CIN 2/3 during the period of April 2017 to March 2018. No data were available on this regard in Sri Lanka during the study period. According to a study done in Egypt among 3600 women who underwent screening with VIA and were subjected to colposcopy-guided biopsy, the prevalence of CIN 1 was found to be 3.3%, and CIN 2/3 was 1.11% [16]. When our prevalence is compared with this study, there were a smaller number of cases diagnosed with CIN 1 and proportionately more cases diagnosed with CIN 2/3 since the biopsy was taken only in those with a swede score of 5 or more in our study, which targeted high-grade lesions only (CIN 2/3).

Acceptability of VIA as a screening test was high since 97.86% of women would recommend VIA to their relatives and friends (N=914). Only six women (0.64%) had heavy bleeding from the biopsy site, and 71 women (7.6%) stated that they had severe pain during the procedure. Considering the benefit of detecting CIN before it evolves into cervical cancer, these minor complications are acceptable.

When assessing the risk factors for cervical cancer in the study population, only 113 women (12.1%) stated that they had been sexually active before the age of 18 years, and only five women (0.53%) stated they had more than one sexual partner. This data may be underestimated, especially with an interviewer-administered questionnaire, because of stigma around sexual practices in Sri Lanka. Three hundred and fifty-four women (37.9%) had five or more pregnancies, including miscarriages, and 269 women (28.8%) were exposed to passive smoking, but none were active smokers, as smoking in women is very rare in Sri Lanka. A significant proportion of women (37.15%, N= 347) complained of at least one symptom of STD, but it cannot be assumed that the prevalence of STD is high in this population, as there is significant overlap of these symptoms with other gynaecological causes. One hundred and eighty-six women (19.9%) used OCP at any time in their lives. The prevalence of use of other contraceptives and their association with pre-neoplastic conditions was not assessed, as most women used more than one contraceptive method.

Among these risk factors, early sexual activity before 18 years (p<0.005), ever usage of oral contraceptive pills (p<0.000), and five or more pregnancies, including miscarriages (p< 0.005), were significantly associated with CIN 1 or more using the chi- squared test. All these factors are well known to increase the risk of cervical carcinoma.

According to a systematic review conducted by Sritipsukho et al., which included 11 studies during the period of 1996-2007, VIA was found to have a pooled estimate of sensitivity of 71.8%, specificity of 79.4%, PPV of 16.7%, and NPV of 99.0% in detecting any CIN 2/3 lesions [17]. Compared to the above result, this study reported a sensitivity of 70.6%, specificity of 74.7%, PPV of 4.9%, and NPV of 99.3% when using a cut-off of CIN 2 or more. Even though the sensitivity, specificity, and NPV in our study were comparable with the systematic review, the PPV was significantly lower than that of the systematic review. It could be due to using a lower threshold for reporting VIA as positive, which will include non-neoplastic changes such as inflammation and immature squamous metaplasia. A higher VIA-positive rate of 26.1% in our study supports this, whereas the acceptable VIA-positive rate is around 8-15% [13]. 

Limitations

This study was conducted in a single study setting. So, it was impossible to collect a sample with heterogeneous ethnic, genetic, or socio-cultural variants, which could be achieved by a clustered randomised sampling technique. As this study was done in an outreach clinic, it was not possible to include all islands of the Velanai MOH area due to the unavailability of safe transport facilities for the colposcope and other equipment. The above-mentioned reasons may affect the representativeness of the study sample. As the data collection was done through an interviewer-administered questionnaire to reduce the number of non-responders, the prevalence of risky sexual practices such as early sexual activity and multiple sexual partners may be unreliable. So, the obtained non-significant association with the number of sexual partners could be due to this information bias.

VIA is not effective in detecting preneoplastic lesions in most postmenopausal women in whom the squamo-columnar junction of the cervix is unreachable for application of acetic acid. In these types of patients, endocervical curettage is needed for further evaluation, and histology or colposcopy can be repeated after a short course of treatment with oestrogens to bring the transformation zone out of the endocervical canal. As our research was done in an outreach clinic as a one-stop procedure, we couldn’t follow these standards, so we have excluded those women with an invisible squamo-columnar junction, which may affect the calculation of test performance through selection bias.

Even though the colposcopy was performed only by a researcher, the screening test was performed by four intern house officers. This may lead to inter-observer errors, and it may affect the test performance. Even though these house officers were trained on VIA, they are less experienced, so they may tend to report more VIA positivity for faint acetowhite areas. This information bias could be the reason for the observed low positive predictive value and high false positive rate in this study and may affect internal validity.

## Conclusions

The prevalence of CIN 2/3 was 1.82% in the study population during April 2017 to March 2018. Further studies in multicentre settings are needed to establish the national prevalence, as this is not yet established. Significant association was noted for early sexual activity before 18 years, ever usage of oral contraceptive pills, and five or more pregnancies, including miscarriages, with CIN 1 or more lesions. Even though VIA has low positive predictive value and relatively high false positive and false negative rates, VIA can be considered as an alternative screening tool for cervical carcinoma in low-resource areas, as it is easily performed and incurs very little expense. The high level of acceptability of VIA in the target population also supports its use as a screening test.

## Appendices

Information sheet (English)

I am Dr. Mathanky Theeparuban, Registrar in Obstetrics and Gynaecology, working in the University Unit, Teaching Hospital, Jaffna. I would like to invite you to participate in the study titled “Effectiveness of visual inspection of acetic acid as a screening test for cervical neoplasia” conducted by me and Dr. K. Muhunthan (VOG), Teaching Hospital, Jaffna. Your voluntary participation in this study is highly appreciated. You are free to refuse to participate or to withdraw from the study at any time. If you decide not to participate or withdraw from the study, you may do so at any time.

Cervical cancer develops from precursor lesions called cervical intraepithelial neoplasia. These lesions can be detected by cervical screening by visual inspection with acetic acid. It is recommended to screen every woman who has been sexually active, after 35 years in at least a 5-year interval, up to 60 years in Sri Lanka. Early detection of premalignant lesions by screening and appropriate treatment will prevent the development of invasive cancer. 

The purpose of this study is to assess the effectiveness and acceptability of screening for cervical cancer by visual inspection with 5% acetic acid (vinegar), in an attempt to reduce the morbidity and mortality associated with cervical cancer. I also like to see the prevalence of preneoplastic conditions and their association with the risk factors for cervical cancer. 

In our study, we planned to screen women in the age group of 35-49 years. After the screening test, you will have an examination by a special magnifying instrument called a colposcope on the same day. If the colposcopy examination is positive, we will take a small tissue sample (biopsy) from your cervix to confirm it with further examination by a histopathologist. These procedures are mostly painless, but you may feel some burning sensation while applying acetic acid. You may have some giddiness, minor bleeding, or vaginal discharge after the procedure. Rarely may you develop heavy bleeding, which can be easily controlled.

After the screening, you will be issued an interviewer-administered questionnaire. The purpose of this questionnaire is to assess your risk for developing cervical cancer and your level of acceptance of this type of screening test. Your contact information will also be obtained to inform you regarding your test results.

Your test results will be informed to you through your MOH/midwife/directly by us, and further follow-up will be arranged. Depending on the result of histology, you may need minor surgery to remove the diseased part of your cervix or a major surgery and/or radiotherapy in TH/Jaffna.

The collected data will be maintained confidentially, and your personal identification data will not be disclosed or published. Data will be stored in a personal computer. After completion of this research, all questionnaires will be discarded safely, and the data in the computer will be confidentially maintained for five years. This data will be used for this research only.

If you have any questions regarding the procedures, you can freely communicate with us. Contact number of principal investigator - 0779004961

Certificate of Consent

I have read the foregoing information, or it has been read to me. I have had the opportunity to ask questions about it and any questions that I have asked were answered to my satisfaction. I have given with sufficient time to come to a decision, and I consent voluntarily to participate in this research.

Name of Participant ..................................................................................

Signature of Participant ..............................................................................

Date ...........................................

Statement of the researcher/ person taking consent

I have completely explained and read out the information sheet to the potential participant to the best of my ability and made sure that the participant understands clearly about the procedures.

I confirm that the individual has not been forced to give consent, and the consent has been given freely and voluntarily.

Name of the person taking the consent .......................................................... 

Signature of the person taking the consent .........................................................

Date ..........................................

Questionnaire: Effectiveness of VIA as a Screening Test for Cervical Intraepithelial Neoplasia

A.     Patient identification details                                       Date …………………………

1.      Serial number:  …………………………….                                  

2.      Patient name: ………………………………………...…          Telephone No:   …….………….…………

3.      Address: …………………………………………………………………………………………………………………….

B.      General characteristics

1.            Date of birth:                                                           Age at last birthday:   ………….

2.            Religion           

a)      Hindu    b) Christian    c)   Muslim     d) Buddhist    e) others………………….

3.            Educational level

a)      Uneducated     b) < Grade 5       c) Grade 6- O/L      d) A/L       e) Higher education

4.            Socio economic status/monthly income

a)      <10,000      b) 10,000-29,999     c) 30,000-49,999      d) >50,000

5.      Marital status

a)      Married    b) Widowed   c) Separated   d) Others…………………….

C.      Past gynaecological history and risk factors

1.      Age at menarche

2.      Date of last menstruation …………………………………….

a)      <12 months ago       b) >12months ago

3.      Age at marriage / first intercourse    

a)      <18     b) >18   

4.      No of lifetime partners .................

5.      Total number of pregnancies & miscarriages

a)      < 5       b) 5 or more

6.      Contraceptive use - (state the duration of use in years)

a)      None b) injectable c) oral d) barrier e) IUCD f) Implant g) LRT h ) others…….

7.      STD History - Do you have / did you have any of following symptoms?

(Use √ if your response is yes; otherwise leave it blank)

a)      Excessive vaginal discharge                                                      

b)      Mal-odorous vaginal discharge

c)      Itching of vulva and vagina

d)      Ulcers in the genitalia

e)      Pain during intercourse

f)       Bleeding during intercourse

g)      Lower abdominal pain

h)      Inter menstrual bleeding

8.      Smoking

a)      Active smoking       b) Passive smoking        c) Nil

9.      Past history of autoimmune disease/ immunosuppressive therapy

a)      No        b) Yes

D.     Acceptability

1. Would you recommend screening to your friends / relatives?

     a) Yes      b) no

2. Did you experience any problems during screening

    (Use √ if your response is yes; otherwise leave it blank)

    a)  Severe bleeding

    b)   Severe pain

Reporting Form of VIA

Modified from the Visual inspection manual, IARC [13].

1. Select appropriate by indicating “√” if present

a) Squamo-columnar junction fully seen

b) Cervical polyp

c) Nabothian follicles

d) Cervicitis

e) Leucoplakia

f) Condyloma

g) Growth

2.      Finding of VIA

a)  Negative     b) Positive       c) Suspicious for invasive cancer

3.      If the VIA is positive, does it extend into endocervical canal?

a) Yes       b) No

4.       If the VIA is positive how many quadrants are involved by aceto-white lesions?

a) Two or less      b) three       c) four

5.      Diagram of VIA finding (Draw the squamocolumnar junction with a dotted line and aceto white areas as a continuous line)

Reporting form for colposcopy and histopathology

Modified from colposcopy reporting format - IARC [14]

Select appropriate by indicating “√“ if present

1.   General assessment

1.1. Squamo-columnar junction   a) Completely seen     b) Partially seen    c) Not visible

1.2. Type of transformation zone       a) Type 1              b) Type 2                c) Type 3

2.      Normal colposcopy findings

a)      Cervical ectropion

b)      Squamous metaplasia

c)      Nabothian cyst

d)      crypts

3.      Abnormal colposcopy findings

Minor changes

a)      Thin acetowhite epithelium

b)      Irregular geographical lesions

c)      Fine mosaic

d)      Fine punctuation

Major changes

e)      Dense acetowhite epithelium

f)       Rapidly appearing aceto whitening

g)      Sharp border

h)      Cuffed crypt openings

i)        Coarse mosaic

j)        Coarse punctation

k)      Inner border sign

l)        Ridge sign

Nonspecific changes

m)   Leucoplakia

n)      Iodine negative epithelium

4.      Findings suspicious for invasive cancer

a)      Atypical vessels

b)      Exophytic growth

c)      Ulceration

d)      Necrosis

5.      Other findings

a)      Lesion extended into cervical canal

b)      Contact bleeding

c)      Purulent cervicitis

d)      Opaque discharge

e)      Yellow discharge

6.      Finding outside the transformation zone ………………………………………………………..………… …………………………………………………………………………………………………………………………….…….

7.      Diagram of colposcopy finding

(Draw the squamocolumnar junction with a dotted line and aceto-white areas or non-Iodine uptake areas as a continuous line, punctuation(P), mosaics(M), atypical vessels(A) and other lesions)

                      VIA                                                                                                       VILI

8.      Swede score ……………………….

**Table 5:** Sweed score

Features at colposcopy	Score		
	0	1	2
Uptake of acetic acid	Nil/ transparent	Shady, milky	Distinct, stern like
Margins & surface	Nil/ diffuse	Sharp but irregular, jagged, geographical satellites	Sharp and even, elevated / cuffing
Vessels	Fine, regular	Absent	Coarse or atypical
Size of lesion	<5 mm	5-15 mm/ spanning 2 quadrants	>15mm/ spanning 3-4quadrants/ involving endocervix
Iodine staining	Brown	Faint / patchy yellow	Distinct yellow

9.      Final result of colposcopy (Select appropriate by indicating “√”)

a. Unsatisfactory

b. Normal 

c. Inflammation/ infection

d. Leucoplakia

e. Condyloma

f. Low grade CIN (swede score <5)

g. High grade CIN (swede score 5-10)

h. Invasive cancer

i. Others, specify……………………………………… 

10.  Site of punch biopsy (record in the diagram with an “X” mark)

11.  Histopathology report

a) Negative for CIN or invasive cancer                   b) CIN-ǀ

c)   CIN-ǀǀ                      d)   CIN-ǀǀǀ                               d)     Invasive carcinoma
